# Letter to the Editor concerning “Altered chondrocyte apoptosis status in developmental hip dysplasia in rabbits”

**DOI:** 10.4274/balkanmedj.2016.1876

**Published:** 2017-09-29

**Authors:** Feridun Çilli

**Affiliations:** 1 Department of Orthopaedic Surgery, Acıbadem University School of Medicine, İstanbul, Turkey

To the Editor,

I have read the paper with interest “Altered Chondrocyte Apoptosis Status in Developmental Dysplasia in Rabbits” by Wei et al. (1) in the current issue of the Balkan Medical Journal. Wei et al. (1) concluded that ‘prolonged immobilisation of a rabbit’s hip caused chondrocyte apoptosis, but that a reduction of the hip joint may avoid apoptosis, thus preventing secondary osteoarthritis. On this point I agree with the authors.

There are many factors involved in the development of joint degeneration, including prolonged joint dislocation and immobilisation. Prolonged joint dislocation, as seen in developmental dysplasia of the hip, is a mechanical reason for cartilage degradation. Developmental dysplasia of the hip places abnormal physical stresses on the hip joint, which eventually results in degradation of the cartilage tissue. Immobilisation, even of congruent joints, is another important factor that has negative effects on chondrocyte metabolism. It has been shown that immobilisation promotes atrophy of the articular cartilage in humans (2,3) and in animal models (4,5). All of the negative effects of prolonged dislocation and immobilisation start with chondrocyte apoptosis at the cellular level.

Developmental dysplasia of the hip patients may have just dysplasia, subluxation or dislocation, but no joint immobilisation. In this study, the rabbits in the groups had both dislocation and immobilisation at the same time. Thus, the resultant chondrocyte apoptosis could be accepted as the total negative effect of both dislocation and immobilisation. The conclusion of this study would be more appropriate if an emphasis was placed on the detrimental effects of both factors combined on chondrocyte metabolism. The use of an animal model with only immobilisation but without dislocation would allow for a comparison of the effects of immobilisation and dislocation on chondrocyte apoptosis.
